# Identification of health-related problems in youth: a mixed methods feasibility study evaluating the Youth Health Report System

**DOI:** 10.1186/s12911-024-02465-8

**Published:** 2024-03-05

**Authors:** Petra V. Lostelius, Catharina Gustavsson, Eva Thors Adolfsson, Anne Söderlund, Åsa Revenäs, Ann-Britt Zakrisson, Magdalena Mattebo

**Affiliations:** 1 Centre for Innovation, Research and Education, Region Västmanland, Västmanland Hospital Västerås, Västerås, Sweden; 2 School of Health, Care and Social Welfare, Mälardalen University, Västerås, Sweden; 3 Clinic for Pain Rehabilitation Västmanland, Region Västmanland, Västerås, Sweden; 4 Centre for Clinical Research Dalarna, Uppsala University, Falun, Sweden; 5 School of Health and Welfare, Dalarna University, Falun, Sweden; 6 Department of Public Health and Caring Sciences, Uppsala University, Uppsala, Sweden; 7 Centre for Clinical Research, Region Västmanland– Uppsala University, Västerås, Sweden; 8 Orthopedic Clinic, Västerås Hospital Region Västmanland, Västerås, Sweden; 9 University Health Care Research Center, Faculty of Medicine, and Health, Örebro University, Örebro, Sweden

**Keywords:** Electronic patient-reported outcome, Feasibility study, Health and welfare technology, Medical informatics, Mixed-methods research, Young people, Youth health clinic

## Abstract

**Background:**

Because poor health in youth risk affecting their entry in adulthood, improved methods for their early identification are needed. Health and welfare technology is widely accepted by youth populations, presenting a potential method for identifying their health problems. However, healthcare technology must be evidence-based. Specifically, feasibility studies contribute valuable information prior to more complex effects-based research. The current study assessed the process, resource, management, and scientific feasibility of the Youth Health Report System prototype, developed within a youth health clinic context in advance of an intervention study.

**Methods:**

This mixed-methods feasibility study was conducted in a clinical setting. The process, resource, management, and scientific feasibility of the Youth Health Report System were investigated, as recommended in the literature. Participants were youth aged 16–23 years old, attending a youth health clinic, and healthcare professionals from three clinics. The youth participants used their smart phones to respond to Youth Health Report System health questions and healthcare professionals used their computer to access the results and for registration system entries. Qualitative data were collected from interviews with healthcare professionals, which were described with thematic analysis. Youth participants’ quantitative Youth Health Report System data were analyzed for descriptive statistics.

**Results:**

Feasibility analysis of qualitative data from interviews with 11 healthcare professionals resulted in three themes: *We expected it could be hard*; *Information and routines helped but time was an issue*; and *The electronic case report form was valuable in the health assessment.* Qualitative data were collected from the Youth Health Report System. A total of 54 youth participants completed the evaluation questionnaire, and healthcare professionals retrieved information from, and made post-appointment system entries. Quantitative results revealed few missing items and acceptable data variability. An assessment template of merged qualitative and quantitative data guided a consensus discussion among the researchers, resulting in acceptable feasibility.

**Conclusions:**

The process-, resource-, management-, and scientific feasibility aspects were acceptable, with some modifications, strengthening the potential for a successful Youth Health Report System intervention study.

**Supplementary Information:**

The online version contains supplementary material available at 10.1186/s12911-024-02465-8.

## Background

Although young people in Western countries are relatively healthy [1], poor lifestyle and mental health issues decrease their well-being [1, 2]. Since the 1990s, and especially the last ten years, mental health issues have increased in children, adolescents, and young adults [3], and due to the COVID-19 pandemic, adolescents have become more likely to suffer from depression and anxiety [4]. Early mental health issue onset also poses risks to long-term quality of life [5, 6], as strong evidence links psychosocial problems during youth with poor mental health during adulthood [2, 7]. In the United States, screening for depression and suicide risk in adolescents 12–18 years old, is recommended [8], and for anxiety in ages 8–18 [9]. Broad screening for mental health issues has potential to lead to better health and prevent unnecessary morbidity and mortality [10]. This evidence gives, that there is need for early identification of health and health-related problems in young people, with methods adapted to their ways of living. The age span from childhood through adolescence to young people can be defined in many ways. Best describing this study’s population of young people is the United Nations’ definition ‘youth’ for 15 to 24 years old people [11].

In Sweden, mental health issues and psychosomatic symptoms (e.g., headache, sleeping problems, dizziness) have increased [12]. For the youth population, health and welfare technology (HWT) has been used to identify psychosocial issues [13–15] and improve health equity [16], since the HWT tool has been found to facilitate the sharing of feelings and increased the reporting of psychosocial issues [17]. In 2016, Sweden adopted the vision of becoming world-leading in digitalization and HWT by 2025, with aims to increase individuals’ health, reach health equity, increase independence, and ensure all citizens’ participation in society [18]. HWT use is developing in many health areas, targeting different populations [19–21].

HWT in the form of digital surveys can be effective to illuminate psychosocial issues [22]. Despite foundational needs for evidence testing of effectiveness for use of HWT [23], in the healthcare setting the evidence of effectiveness of HWT is often missing [24]. Thus, this third study in a participatory research project attempts to explore evidence for feasibility of a HWT in the setting of youth health clinics (YHC). Previously, two studies have been reported, describing the development and usability evaluation of an electronic health assessment tool [25, 26].

The objective with the current study was to assess the process, resources, management, and scientific feasibility aspects of a Youth Health Report System prototype, developed within a YHC clinical context, in advance of an intervention Stepped Wedge Cluster Randomized Trial. Specifically, within each feasibility aspect, the intention was to answer the questions:

1. Process: Is the recruitment potential and process adequate for a successful future intervention study?
2. Resources: Is there adequate resources to administer the study procedure as planned? Is the Information Technology (IT)-platform accessible to provide the possibility to deliver the intervention?
3. Management: Is it possible to access data for use in the health assessment? Is it possible to understand the reported health data for use in the health assessment?
4. Scientific: Do the electronic evaluation questionnaire collected data have adequate quality?

## Methods

### Study design

This feasibility study [23, 27, 28], in which participants were assigned to the intervention or control group, used mixed methods to collect and analyze data [29]. Study reporting was guided by the Consolidated Standards of Reporting Trials (CONSORT) 2010 statement on randomized pilot and feasibility trials [30] and by Thabane et al. [27]. The study was registered (ISRCTN23855544).

### Setting

In Sweden, healthcare is governed within regions. In all regions, YHCs offer first-line health services for those aged 13–25 years. YHCs are multi-professionally staffed, by midwives and physicians who primarily promote sexual health, and by healthcare counsellors and (occasionally) psychologists who promote mental health. Some YHCs also offer dietitian and physiotherapist counselling. YHCs can be run by the region, municipality, privately, or a combination of these actors, and each is led by a manager who has access to a consulting physician [31].

### Participants

This feasibility study included participants from two YHCs in two regions of central Sweden, including a small rural clinic, a midsize urban clinic. The patient inclusion criteria were age 16–23, fluent in Swedish, and having a health-related YHC appointment. Based on previous years’ estimates of YHC visits, the target sample was 500 patients. We expected that 30% of the 500 would respond to the digital survey, based on the clinical experience of YHC healthcare professionals of youth survey engagement and that a realistic expectation for online surveys is 35–50% [32]. Further, to cover the cultural dimensions [33] of the YHC professionals, regarding profession and computer literacy, we sought to include at least six healthcare professionals.

### Youth Health Report System

**Fig. 1:**
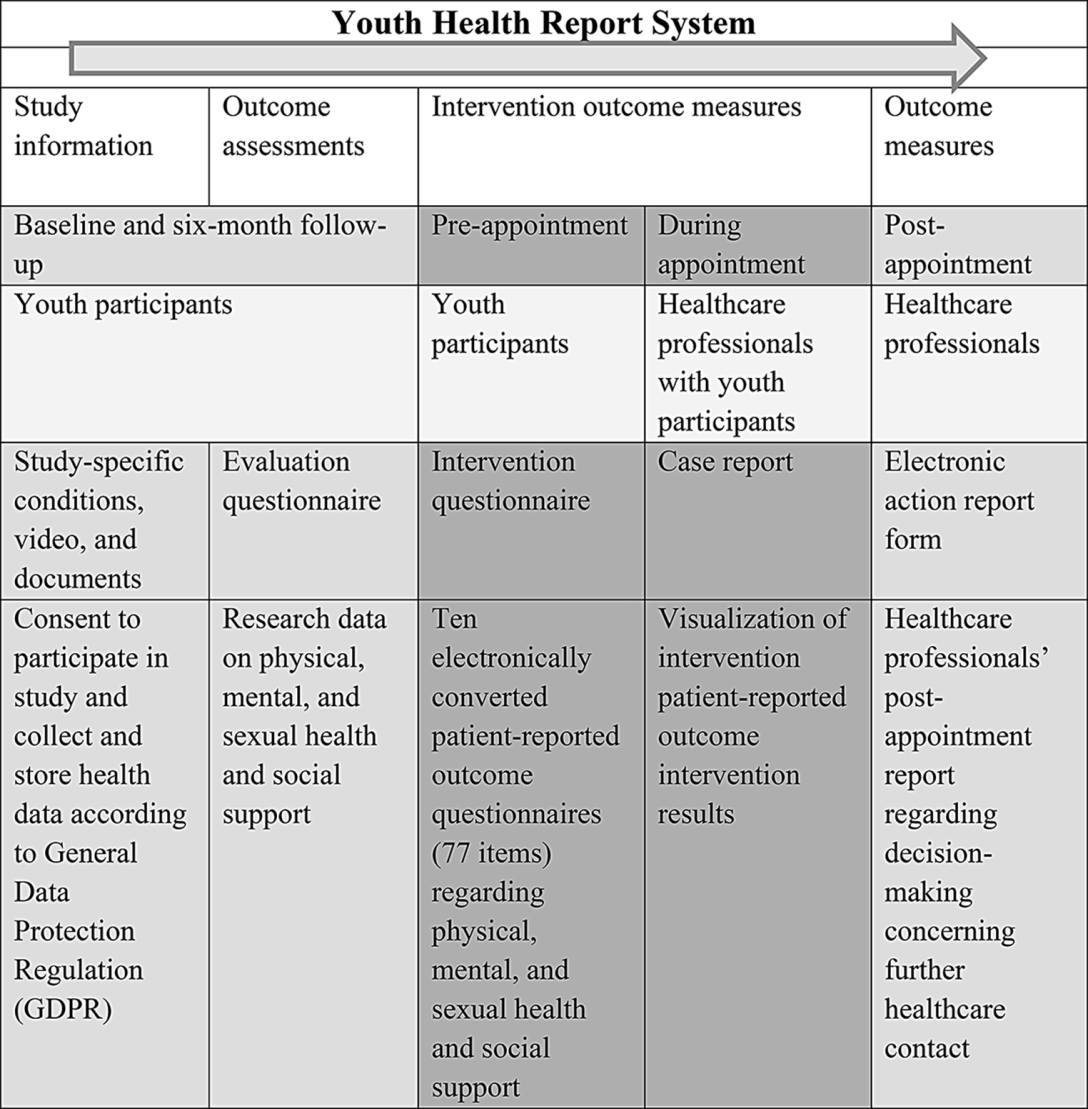
Youth Health Report System features and their uses

The research group previously developed and described the Youth Health Report System (Fig. 1) [26]. Briefly, the prototype system includes study and digital consent information, followed by an electronic evaluation questionnaire (henceforth, ‘evaluation questionnaire’). The evaluation questionnaire was used to collect research data at baseline and six-month follow-up on participant background, mental, physical, and sexual health, and social support. There were three intervention materials: the patient electronic health report form (henceforth, ‘intervention questionnaire’); the patient electronic case report form (henceforth, ‘case report’); and the healthcare professional electronic action report form (for reporting planned post-healthcare assessment actions).

### Procedure

**Fig. 2:**
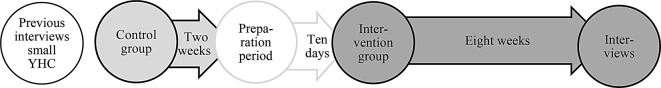
Data collection points starting with the previous interviews, the control group, and intervention group periods

The healthcare professionals at the small YHC participated in the usability evaluation and were thus interviewed previously. They were informed about the study and gave verbal and written consent. Figure 2 provides an overview of the full study procedure.

At the midsize YHC, youth participants were recruited from April 6 through June 23, 2021. Due to the forthcoming summer vacation, and hence fewer YHC visits, the planned 15-week study period necessarily became 12 weeks, including a preparation phase before beginning the intervention phase. Due to the COVID-19 pandemic, YHC drop-in visits were prohibited, and healthcare visits were generally sparse, resulting in a very limited number of potential youth participants. Youth participants were allocated to either the control or intervention group (Fig. 2) based on the timing of their YHC appointment. These steps were designed to mimic a Stepped Wedge Cluster Randomized Trial.

When making a YHC appointment, youth participants were registered (with their name, social security number, and smartphone number) in the information technology platform (IT-platform) by a healthcare professional. The IT-platform, with secure data collection according to the General Data Protection Regulation (GDPR) [34], then sent the patient a short message service (SMS) with study information and digital consent to the young person’s smart phone. After receiving the information, it was demanded to consent participation in the study by clicking ‘yes’. When consenting, the youth participants gained access to and responded to questions about their health and background characteristics in the evaluation questionnaire. Those who did not complete the survey received a reminder SMS the same day and again three days later.

The healthcare professionals at the midsize YHC, who performed the feasibility study procedure, provided informed verbal consent, and later mailed their written consent to the first author.

### Control group

The study began with a two-week control phase (Fig. 2). All youth participants visiting the YHCs during this period received an SMS with study information, a digital link to video information (2 min, 6 s), a written information document, and digital consent. The video contained short information about the study purpose, the research principal, the research procedure, if participation concerned control- or intervention group, what kind of questions were included, that participation was voluntary and that regret to participate would not affect the reception at the YHC. Further, the video included information on the General Data Protection Regulation (GDPR), how data were stored, and who to turn to with any questions. The written information document contained this information in detail. Upon consenting, the patient gained access to the evaluation questionnaire via their smartphone and responded prior to their YHC appointment. Youth participants in the control group were offered the usual care.

Between the control and intervention groups, a 10-day period was used to prepare the YHC healthcare professionals for the intervention and to finalize intervention procedures. This duration was determined with input from the YHC manager.

### Intervention group

The intervention group was recruited for eight weeks (Fig. 2). All youth participants with YHC appointments during this period were offered the intervention. The intervention group received the same information as the control group, with an addition about the intervention questionnaire health questions, and the digital consent. Upon consenting and completing the evaluation questionnaire, they received a second SMS with the intervention questionnaire prototype. The healthcare professional then used the case report in their health assessment during the appointment.

The intervention had two parts: the intervention questionnaire and the case report (Table 1). The intervention questionnaire contained background questions and the reason for the YHC visit, followed by lifestyle and health questions using reliable, validated patient-reported outcome questionnaires. At the end of each health area, self-efficacy questions assessed first, the patient’s perceived need to make changes to improve their health and/or reduce health risks, and second, the patient’s behavior change self-efficacy [26].

**Table 1:** Characteristics of the instruments included in the evaluation questionnaire

Health area	Instrument	Validity	Item(s)	Response options
Mental health	SWEMWBS	Youth [53] Swedish [54]	Seven statements to examine psychological well-being	Five-point (p) Likert scale: always (1p) / often (2p) / sometimes (3p) / rarely (4p) / not at all (5p) Highest possible score is 35 p. High points indicate high positive mental well-being
	SOMS-7	Swedish, working age [55]	Measures the experience of mastering life with five negatively and two positively formulated statements	Responses on a four-point Likert scale. Negatively formulated response options are reverse-scored. Scores range from 7–28 p, with higher scores indicating higher ability to manage life
Physical health	IPAQ-SF	Swedish 18–65 years olds [38]	Measures physical activity (working, transportation, housework, gardening, leisure time, and planned physical exercise) and sedentary time for the past seven days	The activities provide a score that sums to physical activity time and how often each is performed, expressed as Metabolic Energy Turnover
Sexual health	Self-efficacy questions, self-composed according to Bandura [40]		Subjective rating of self-efficacy for healthy sexual behavior concerning protection from sexually transmitted disease, unwanted pregnancy, and alcohol consumption connected with having sex	Response options range from 0 (not at all sure) to 100 (completely sure). Questions and response options are designed according to Bandura’s guide to constructing scales
Social support	OSSS-3	Working age [56]	Measures social functioning using three questions regarding the number of people the respondent feels close to, interest and concern that others have for the respondent, and how easy it is to get practical help from others	Total score ranges from 3–14 (3–8 = poor support, 9–11 = moderate support, 12–14 = strong support)
	MMQoL	Youth [36] Swedish [57]	Eight statements concerning friendship relationships	Each statement has five response options: Very true / Fairly true / Neither true nor false / Not particularly true / Not at all true

**Fig. 3:**
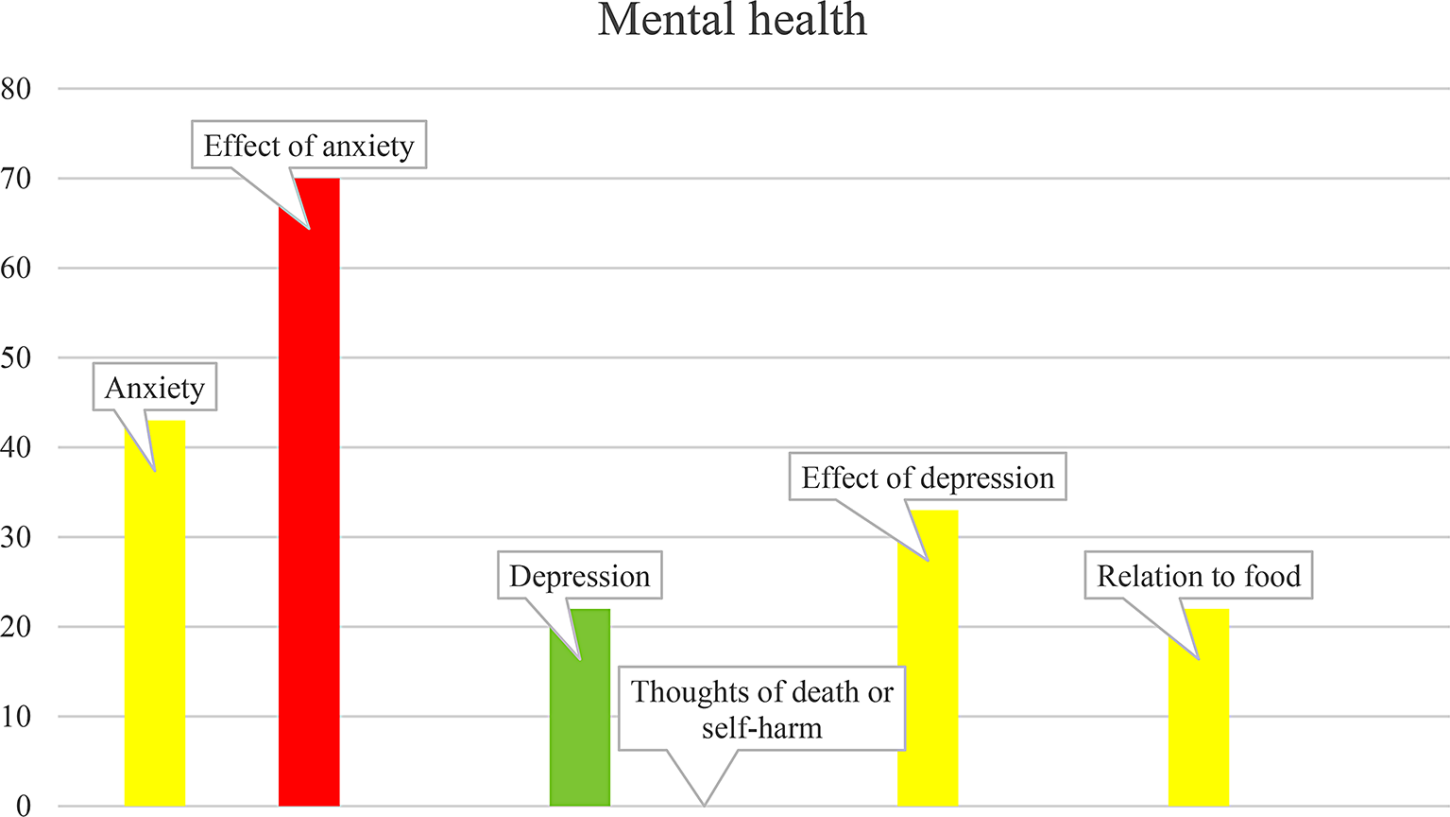
Case report example, with each health risk indicated by color, subject, and score

Intervention questionnaire responses were then compiled in the IT-platform to form the case report, which displayed graphs of health and/or health risk levels in green (good health/low risk), yellow (increased poor health risk/risky health behavior), and red (apparent poor health risk/risky health behavior) as a health status overview for the YHC healthcare professional. An extract from the case report data is displayed in Fig. 3. When possible, the levels were based on the questionnaire’s instructions; otherwise, the research group determined cutoff scores based on clinical experience and expert advice. The case report also included text descriptions after the graphs.

The YHC healthcare professionals were instructed on reviewing the case report on the IT-platform, interpreting its graphs, and using it as background information during the patient health assessment. Other than confirming that the patient’s responses had been received, each healthcare professional was at liberty to use the case report in their assessment (during that appointment or a future one).

### Data collection

To assess the process (recruitment potential), resource (study administration, IT-platform satisfaction), and management (data accessibility, case report interpretation) feasibility, we adapted the Thabane et al. classification system [27].

### Qualitative data

Qualitative data were used to explore process (recruitment potential), resource (study administration, IT-platform satisfaction), and management (data accessibility, case report interpretation) feasibility.

From a previous study in the project [26], unanalysed data from interviews with YHC healthcare professionals, were also used to solicit healthcare professionals’ opinions. Data from the current study were collected in focus groups with the midsize YHC healthcare professionals. Due to COVID-19 restrictions, the interviews were conducted online. The first author performed all interviews, and one co-author (ÅR) participated in two interviews.

### Quantitative data

Quantitative data for evaluating process (recruitment potential) and scientific (data variance, missing items) feasibility were collected from the IT-platform and evaluation questionnaire. Patient background (age, biological sex, living situation, marital status, country of birth, employment, and education level) and health data were also collected from the IT-platform. Mental health items were from: the Short Warwick–Edinburgh Mental Well-Being Scale (SWEMWBS) [35]; The Minneapolis-Manchester Quality of Life instrument (MMQoL) [36]; and the Sense of Mastery Scale (SOMS-7) [37]. Physical health items were from the International Physical Activity Questionnaire (IPAQ) [38]. Sexual health items were three self-efficacy questions [39, 40]. Social support items were from the Oslo 3-item Social Support Scale (OSSS-3) [41]. Table 1 provides a description of the evaluation questionnaire content, health questions, response options, and references to studies of validity.

### Data analysis

#### Qualitative data

Qualitative data analysis of the focus group and individual interviews were performed using theoretical thematic analysis [42].

#### Quantitative data

Quantitative data analyses were performed in Microsoft Office 365 - applications for business; Excel version 15601.20660, and in cooperation with co-author MM. These analyses included summary descriptive statistics for scientific feasibility. The feasibility assessment procedure is presented in Table 2.

**Table 2:** Data analyses for youth health clinic feasibility assessments

Feasibility	Data collection method	Inquiry	Measures	Feasibility assessment
Process				Assessment of whether criteria were met
Recruitment potential	Interviews and evaluation questionnaire	Is sufficient participant recruitment feasible for the study?	Percentage of eligible youth participants recruited and explored in interviews	YHC managers and research team estimated that 30% of YHC visitors can be considered potential participants. The research group will consider quantitative and qualitative data in their overall judgment of recruitment feasibility
Resources				
Study administration	Interviews	Is the study procedure executed as intended?	Exploration of understanding, and time and effort acceptability, of the Information Technology-platform (IT-platform), including the education materials (from qualitative interviews with midsize YHC healthcare professionals)	The research group will consider qualitative data in their overall judgment of study procedure feasibility
IT-platform accessibility	Interviews	Is the intervention delivered as intended?	Exploration of understanding and acceptability of working with the IT-platform (from qualitative interviews with midsize YHC healthcare professionals)	The research group will consider qualitative data in their overall judgment of whether the IT-platform accessibility is feasible for delivering the intervention
	Interviews	Were there any technical problems obstructing the feasibility of the Stepped Wedge Cluster Randomized Trial? If so, what?	Exploration of the feasibility of progressing with the Stepped Wedge Cluster Randomized Trial (from qualitative interviews with midsize YHC healthcare professionals)	The research group will consider qualitative data in determining whether any obstructions occurred, and will consider these in their overall judgment
Management				
Data accessibility	Interviews	Was access to the case report and action registration form in the IT-platform acceptable?	Exploration of understanding and acceptability of accessing data from the IT-platform (from qualitative interviews with midsize YHC healthcare professionals)	The research group will consider qualitative data in making their overall judgment of whether accessibility of the case report and action registration form were acceptable
Case report interpretation	Interviews	Was the healthcare professionals’ case report interpretation satisfactory?	Exploration of understanding and interpretation acceptability of using the case report (from qualitative interviews with midsize and small YHC healthcare professionals)	The research group will consider qualitative data in making their overall judgment of whether the case report and its interpretation were acceptable
Secondary outcomes				
Scientific feasibility				
Data variance	Evaluation questionnaire	Were there any ceiling or floor effects? Was data variance acceptable?	Frequency distributions	The research group will make their overall judgment based on the quantitative data
Missing items	Evaluation questionnaire	Was the number of items lacking responses acceptable?	Frequency distributions	The research group will consider the quantitative data in their judgment of missing data acceptability

## Results

**Fig. 4:**
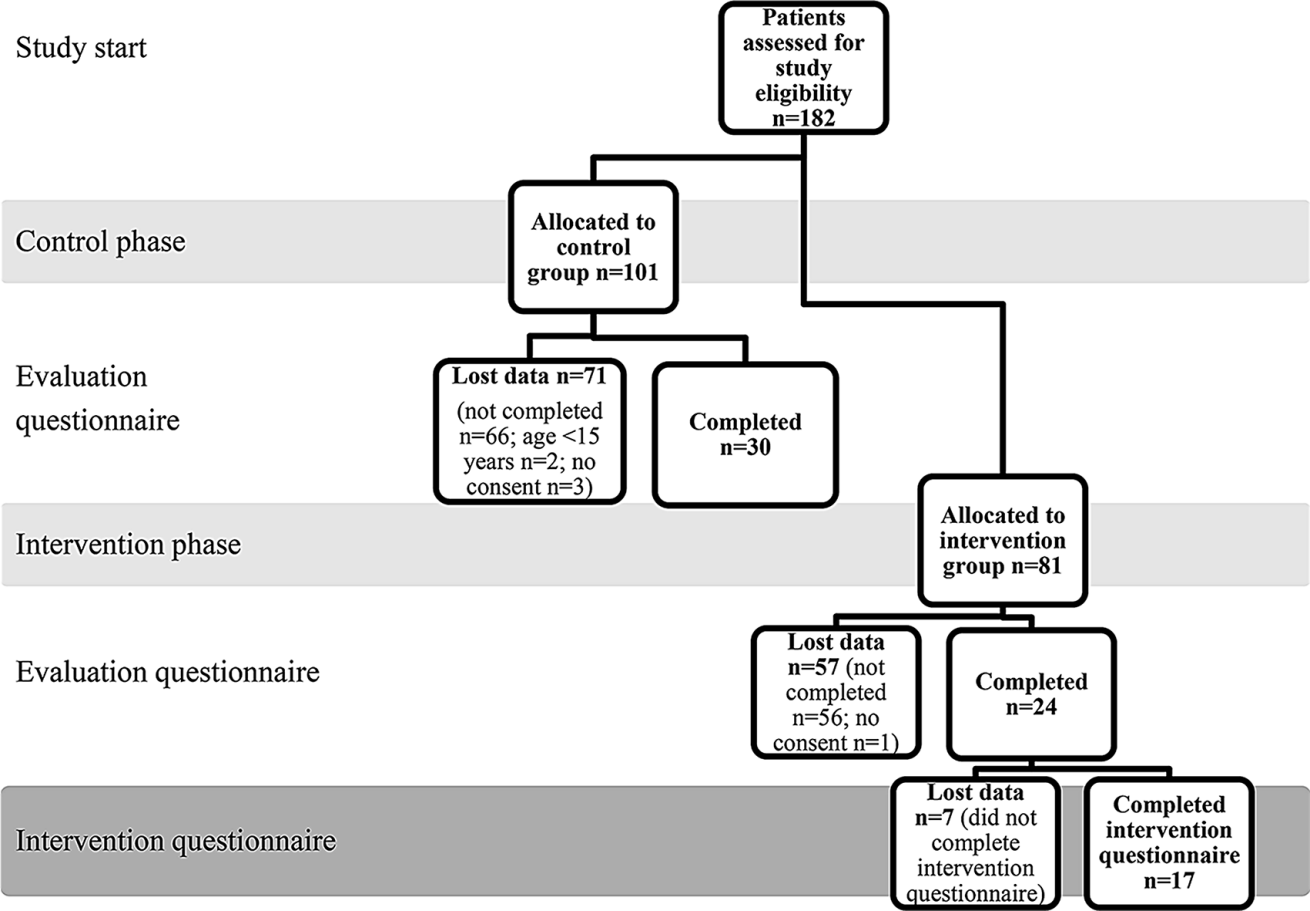
Flow diagram of study enrollment, dropout, and questionnaire completion

In total, 182 youth participants with a YHC appointment were assessed for study eligibility to the feasibility study, among whom 101 visited the midsize YHC during the control phase and 81 during the intervention phase. A total of 17 youth participants completed the intervention questionnaire and had a case report generated for use by the healthcare professional and patient during the patient’s health assessment. Figure 4 is a participant flowchart for the control and intervention groups.

### Participant characteristics

A total of 54 participants in the control and intervention groups completed the evaluation questionnaire. They were predominantly female (89.4%), with a mean age of 18.4 years, born in Sweden (97.9%), and living with parent/guardian (72.3%). Some participants had current contact with other health services (25.5%), mostly for help with mental health (53.8%) (Table 3). There were no significant differences between the intervention and control groups at baseline.

**Table 3:** Participating youth’s background characteristics

Characteristics	Intervention (*n* = 24)	Control (*n* = 30)	Total (*n* = 54)
Age (years), mean (standard deviation [SD])	18.38 (2.23)	18.47 (2.36)	18.38 (2.31)
Female, n (%)	14 (82.35)	28 (93.33)	42 (89.36)
Born in Sweden, n (%)	17 (100)	26 (86.7)	46 (97.87)
Living with parent/guardian, n (%)	12 (70.59)	22 (73.3)	34 (72.34)
Student, any level, n (%)	12 (70.59)	23 (76.7)	39 (74.47)
High school student, n (%)	8 (47.06)	15 (50)	23 (48.94)
Working, n (%)	3 (17.65)	5 (16.7)	8 (17.02)
Currant contact with other health service	5 (29.41)	8 (26.7)	13 (27.66)

Eleven healthcare professionals from two YHCs participated. Those from the midsize YHC were counsellors (*n* = 3), psychologists (*n* = 3), a midwife (*n* = 1), and an assistant nurse (*n* = 1); those from the small YHC were a counsellor (*n* = 1), a midwife (*n* = 1), and a manager (*n* = 1). One participating healthcare professional was male.

### Qualitative evaluation of process, resource, and management feasibility

**Fig. 5:**
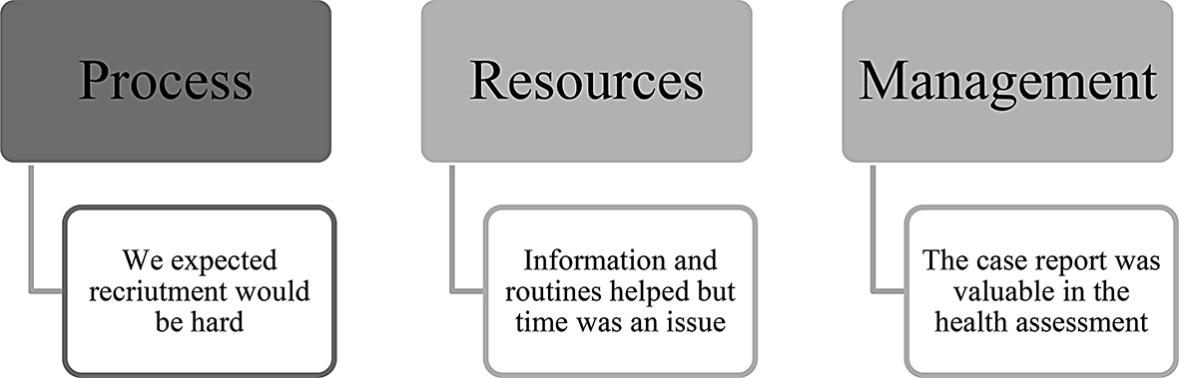
Model of qualitative themes, connected to each feasibility type

Thematic analysis of the healthcare providers’ interviews identified themes for process, resource, and management feasibility (Fig. 5).

#### Process feasibility—recruitment potential

##### We expected recruitment would be hard

The slow recruitment rate confirmed YHC healthcare professionals’ previous experiences and was a study concern. They discussed the potential solution of rewarding youth participants who participated, as well as the ethical dilemma of doing so.

#### Resource feasibility—study administration, IT-platform satisfaction

##### Information and routines helped, but time was an issue

YHC healthcare professionals found the video, written education material, and online educational meetings helpful for understanding how to use the IT-platform. They pointed out the need for creating routines to make tasks smoother. Regarding finding time for youth participants to respond to the intervention questionnaire in a future trial study, they discussed opportunities and challenges (e.g., including the health assessment response time).

#### Management feasibility—data accessibility, case report interpretation

##### The case report was valuable in the health assessment

YHC healthcare professionals found the case report information useful in the health assessment context and thought it could also be used in follow-up assessments. Those from both YHCs identified both benefits and challenges to using the case report. They were positive regarding its health information and reported gaining new knowledge from its use. The case report histogram (Fig. 2) colors were easily understood, and the healthcare professionals appreciated that the text responses enhanced their understanding of the histograms. They believed the case report could be used for follow-up health assessments. However, some were unable to access the case report histograms, and thus based their assessment on only the text responses. They also worried that the case report might increase their workload. It was expressed that the case report may be more useful for midwives than for psychologists and counsellors, based on the local YHCs’ protocols and the self-developed health assessment used by the latter professionals.

### Quantitative evaluation of process and scientific feasibility

#### Process feasibility—recruitment potential

Approximately 26% of the youth participants invited to participate agreed to do so.

#### Scientific feasibility—data variance, missing items

A total of 54 youth participants in the control and intervention groups responded to the evaluation questionnaires. Four of six patient-reported outcome questionnaires had missing items. The number of missing items in the evaluation questionnaire was low overall, ranging from 0 to < 5%. Table 4 provides an overview of each questionnaire’s numbers of items, possible responses, and data missing. Overall, the patient-reported outcome questionnaire data were normally distributed.

**Table 4:** Patient-reported outcome questionnaires and their numbers of items, possible responses, and missing data

Patient-reported outcome questionnaire	Number of items	Number of possible questionnaire item responses	Number of missing data
SWEMWBS	7	378	1
IPAQ	3	162	8
Sexual Health Self-efficacy	3	162	1
SOMS-7	7	378	14
MMQoL	8	432	0
OSSS-3	3	162	0

#### Merging qualitative and quantitative data

The qualitative and quantitative data sets were merged through a research group discussion. Overall, the criteria for process (recruitment potential), resource (study administration, IT-platform satisfaction), management (data accessibility, case study interpretation), and scientific (data variance, missing items) feasibility (Table 5) were considered fulfilled. Potential improvements were also recognized. For example, there was an apparent need for the research group to offer the healthcare professionals supportive routines for using the IT-platform and accessing the case report. Overall, progression to a Stepped Wedge Cluster Randomized Trial was considered feasible, with some modifications. The merged feasibility results and modification comments are presented in Table 5.

**Table 5:** Merged qualitative and quantitative results, leading to feasibility evaluation and comments

Feasibility	Data type	Feasibility criteria	Results	Whether feasibility criterion was met	Comment
Process					
Recruitment potential	Quantitative and qualitative	30% of eligible youth expected to participate	26% of eligible youth participated	YES Recruitment was near target (30%)	
Resources					
Study administration	Qualitative	When using the Information Technology platform (IT-platform), is the education material understandable and the study procedure manageable, from the perspective of healthcare professionals’ time and effort?	The educational live sessions made it easier and reminding videos and written instructions good	YES The education material was helpful	Discuss YHC work planning, to ensure time for the health assessment conversation
IT-platform accessibility	Qualitative	Is working with the IT-platform acceptable? Were there any technical problems obstructing its use? If so, what?	Using the IT-platform was quick and simple No technical problems were reported	YES	Developing routines was recommended
Management					
Data accessibility	Qualitative	Was accessing the case report and action registration form in the IT-platform acceptable?	The case report histograms were not found and used by everyone	YES The text answers also provided needed information	Contact the healthcare professionals with reminders and provide support
Case report interpretation	Qualitative	Was the healthcare professionals’ interpretation of the case report satisfactory?	The case report revealed new information and was easily understood	YES Text answers added detailed histogram information	
Scientific					
Data variance	Qualitative	Were there any ceiling or floor effects? Was data variance acceptable?	No ceiling or floor effects were detected	YES Descriptive statistics showed acceptable data variance	
Missing items		Was the number of missing items acceptable?	Items missing from 4 of 6 instruments: SWEMWBS (1); IPAQ (8); Sexual Health Self-efficacy (3); SOMS-7 (14)	YES The number of missing items was acceptable at < 5%	

## Discussion

The findings in this study show that the Youth Health Report System, including the intervention questionnaire and case report, was feasible regarding process (recruitment potential), resources (study administration, IT-platform satisfaction), and management (data accessibility, case report interpretation). Scientific feasibility of the questionnaire (data variance, missing items) was also found to be acceptable. Some issues were also identified for improvement before proceeding with future studies, including finding time for youth participants to respond to the evaluation and intervention questionnaires, time for discussing the case report during the health assessment, and ensuring that healthcare professionals feel secure with extracting the case report from the IT-platform.

The health care professionals raised concerns about difficulties recruiting youth participants, based on previous experiences. The challenge of engaging youth in research may be their lack of availability during school hours and holidays, or that they are unable to make decisions autonomously [43]. Recruitment was also challenged by limited YHC visits during the COVID-19 pandemic. However, among the 182 youth participants asked to participate, 26% consented and participated. This corresponds with HWT response rates in other healthcare settings [44], indicating that our recruitment procedure is feasible. Compared with the study protocol registration, the executed study was shorter in duration than planned, because fewer service staff were available during the summer. This further affected recruitment: The control phase was two weeks instead of three, and the intervention phase was eight weeks instead of 12. There was also a 10-day preparation between the control and intervention phases. Overall, the study was performed over 12 weeks instead of the planned 15 weeks, which may also have affected recruitment. Overall, process feasibility was considered good for future studies.

The COVID-19 pandemic time period was unique. All healthcare professional education sessions were online but seemed to work well. However, the number of young people who came to the YHC for support was limited due to national social restriction, and hence affected the recruitment of youth participants. Also, the number of digital visits to the YHC increased rapidly. This may have posed a challenge for the healthcare professionals who lacked confidence in using computers and thus made it more difficult to use the case report, compared to regular in-person-visits. However, an open-minded and problem-solving approach to the rapid technological changes in healthcare was also recognized to be potentially positive [45]. Furthermore, there is no certain way to know if and how the health assessment conversation was affected, because there were no such evaluations made.

Before the control and interventions phase, all healthcare professionals participated in online meetings and were provided with an information video and written material to help them use the IT-platform and perform study-specific tasks. The healthcare professionals reported finding the educational sessions helpful. A scoping review found that technology is integral to modern pedagogy [46]. This indicates that the shift to the digital sessions does not propose a barrier. However, in the current study, some healthcare providers reported being unable to find the case report graphs, and thus used only the text responses. Although this shows a need to support healthcare professionals to develop work routines, it also confirms that the text responses were useful for the health assessment. All considered, the resource feasibility was acceptable.

The healthcare professionals were concerned that their workloads would increase, though they also expressed that this new management method may be helpful. It is known that user expectations influence their willingness to use HWT [47]. A systematic review identified a variety of barriers and facilitators to implementing HWT in older adults and persons with disabilities. YHC healthcare professionals work with young people, but such barriers and facilitators may also apply to their context. Barriers and facilitators were not specifically explored in this study and are thus important for future implementation assessments. Nevertheless, the research group consensus discussion found that management feasibility was acceptable.

Scientific feasibility concerns data variance and missing items. Missing items were found in four of the six patient-reported outcome questionnaires, with < 5% missing items, respectively. No data were missing at the constructive level (i.e., all patient-reported outcome questionnaires had data represented on all items). Although missing data is a risk for biased estimates, it is possible to adjust for this statistically to minimize such risk [48], indicating feasibility for using the patient-reported outcome questionnaires in future studies.

Although developing and using HWT in healthcare is in line with the Swedish vision of becoming world-leading in digitalization and HWT [18], before the intervention questionnaire could be implemented at YHCs, an effectiveness study [24] in the right healthcare context [23] was needed. A potential contextual finding herein was that YHC healthcare professionals found the case report information more beneficial for midwives than for psychologists and counsellors. This finding may not be transferable, because psychologists are not common to YHC healthcare staff and the YHC where these professionals work used a self-developed mental health questionnaire. YHCs are staffed in various ways [49] and thus may not all be equally prepared to conduct mental health assessments. Hence, the intervention questionnaire and case report may be considered useful for all healthcare professionals at other YHCs.

One study limitation was the impossibility of dropout analysis due to the unavailability of records on youth participants who declined to participate. A dropout analysis is important to estimate bias between treatment arms and intervention effects. However, this was not an intervention effect study and thus such information was not critical to any health data results [50]. However, in future studies, it would be valuable to know more about the young people who chose not to participate, and why. Another limitation may be the risk for healthcare professionals to either over-, or under interpret the case report, perhaps due to the many included health questionnaires, or due to the graphs’ colorings. However, the interpretation is intended to take place in collaboration with each youth responding to the questions. Hence, the health professionals need to check of the assessment with the young person to ensure correct interpretation of the response.

This study used a mixed-methods approach with a merged display of qualitative and quantitative results [29]. It is the third in our series of participatory research reports, aimed at developing and evaluating the Youth Health Report System for YHCs [25, 26]. The participants were predominantly female and born in Sweden. Although greater diversity is valuable, the study sample reflected the 90% female YHC patient population [51], indicating transferability to other YHCs.

Challenges to consider when performing a feasibility trial include which results are emphasized. Statistical effects and predictions are often highlighted over feasibility, while the latter is the main goal of pilot/feasibility studies [27]. Planning and reporting this study was structured and strengthened by guidance from Thabane et al. [27] and the CONSORT extension for pilot trials [30].

Future research priorities include testing the effects of the intervention questionnaire and case report, and studying the implementation of the intervention questionnaire, at YHCs.

## Conclusions

Process, resource, management, and scientific feasibility, evaluated through merged qualitative and quantitative data, were acceptable and strengthened the potential for progress in future studies, with modifications. One of the most important emergent modifications was that although they found the case report valuable in the health assessment, healthcare professionals need support for integrating the case report into their clinical work routines.

### Electronic supplementary material

Below is the link to the electronic supplementary material.

Supplementary Material 1

Supplementary Material 2

Supplementary Material 3

Supplementary Material 4

Supplementary Material 5

## Data Availability

Pseudonymized data may be available from the corresponding author upon request and subject to GDPR and Swedish Ethical Review Authority requirements.

## Abbreviations

HWT: health and welfare technology
IT-platform: information technology platform
GDPR: general data protection regulation
SMS: short message service
SWEMWBS: Short Warwick-Edinburg Mental Well-Being Questionnaire
IPAQ: International Physical Activity Questionnaire
OSSS-3: Oslo 3-item Social Support Scale
CONSORT: consolidated standards of reporting trials
YHC: youth health clinic
SD: standard deviation
