# Supplementary material for: Identification of health-related problems in youth: a mixed methods feasibility study evaluating the Youth Health Report System
Source: BMC Med Inform Decis Mak. 2024 Mar 5;24:64. doi: 10.1186/s12911-024-02465-8 (PMC10913260; doi:10.1186/s12911-024-02465-8)
Supplement: Supplementary file 1 — Supplementary Material 1 [file 12911_2024_2465_MOESM1_ESM.docx]

# **Supplementary file 2**

## **Procedure, qualitative data collection and analysis**

### **Introduction of the electronic Health Report Form (case report) to the health care professionals**

The healthcare professionals at the small-size Youth Health Clinic were informed of the study conditions and gave their consent verbally and in writing (Lostelius et al., 2022). Due to the early development phase of the Electronic Health Report Form (called intervention questionnaire) prototype, the healthcare professionals were shown the early version of the intervention questionnaire prototype and the Electronic Case Report Form (called the case report), with information of how they were constructed and intended to be used. The healthcare professionals were provided with two fictive case reports and possible ways to interpret the results was discussed. After that, interviews using a semi-structured interview guide were performed on the intervention questionnaire prototype usability, including the usability of the case report.

Before the control phase, the healthcare professionals at the mid-size Youth Health Clinic, received a 60-minutes Microsoft Teams meeting on how to manage the IT-platform. They were also provided with step-by step instructions in pre-recorded videos and pdf material. To further support the healthcare professionals, the first author offered Teams appointments and was available all working hours, to answer questions.

### **Detailed description on qualitative data collection and analysis**

The interviews ranged between 30-60 minutes and were recorded with the Linear PCM Voice Recorder DDR-5300. The interview guide contained questions regarding IT-platform and the case report for the health assessment. To enrich the data on using the case report, the non-published data from the previous study (Lostelius et al., 2022) at the small-size Youth Health Clinic, consisting of three individual interviews with healthcare professionals and the manager, was included.

A semantic approach towards the data, intended to describe the data by organizing the codes and then interpret their meaning. Familiarization with the data started by the first author transcribing the interviews. The transcriptions were read several times. Features corresponding to the research questions were given initial codes. The relevant codes were typed, printed, and cut out to small paper pieces, movable for placing and re-placing them where they best belonged in potential themes. At the same time, there was an ongoing search for an overarching theme. To strengthen the trustworthiness of the analysis process, the authors were helped by the guiding article on Thematic Analysis (Braun & Clarke, 2006). In practice, one transcription was simultaneously coded and gathered to initial themes by the first author and a co-writer A-BZ. The coding was compared and discussed until a mutual understanding on the coding had been reached. After reviewing the initial coding and themes, the first author proceeded with the analysis for the second and third transcripts. The themes were considered for internal bonding similarities, and external differences to other theme codes, separating each theme from another. For this, the coded data extracts were consulted. Each theme was also considered in relation to the entire data set. To further strengthen the trustworthiness, the first author and several of the co-authors (A-BZ, MM, ETA, AS, ÅR) were involved in reviewing the themes and refining them by clarifying their meaning. Examples of the analysis process are displayed in the Table below.

Examples of the process from data extract to code.

| Data extract | Coded for | Feasibility aspect |
| --- | --- | --- |
| It is a difficult target group to motivate… we have developed other questionnaires and other surveys – getting them done is a problem. The response rate is very low, unfortunately | 1. Difficult to motivate the target group  2. The response rate is overall low | Process |
| It´s all right once you get into it. You have to get to know it better | 1. It´s all right  2. Get to know it better | Resources |
| I think it has been nice that everything has come up so I could just scroll through it and see everything compiled…yes, I think it gave a very clear picture before the visit, what the person wants to talk about. And then it was interesting to see if it agreed with the reason for the visit … and, what we actually talked about during the visit. So I could still have it in the back of my mind, the areas important to the person and really want to get them in during the visit. I thought it helped go give the conversation a little nudge in that direction | 1. Wants an immediate summary of the answers, overall and in detail  2. Gave a clear picture of what the person wanted to talk about  3. Helped give the conversation a nudge in the right direction | Management |

## **References**

Braun, V., & Clarke, V. (2006). Using thematic analysis in psychology. *Qualitative research in psychology*, *3*(2), 77-101. https://doi.org/10.1191/1478088706qp063oa

Lostelius, P., Mattebo, M., Thors Adolfsson, E. S., A. , Andersén, M., Vadlin, S., & Revenäs, Å. (2022). *Development and usability evaluation of an Electronic Health Report Form to assess health in young people: a mixed-methods approach*.
