# Supplementary material for: Identification of health-related problems in youth: a mixed methods feasibility study evaluating the Youth Health Report System
Source: BMC Med Inform Decis Mak. 2024 Mar 5;24:64. doi: 10.1186/s12911-024-02465-8 (PMC10913260; doi:10.1186/s12911-024-02465-8)
Supplement: Supplementary file 2 — Supplementary Material 2 [file 12911_2024_2465_MOESM2_ESM.docx]

# **Supplementary file 1**

## **Description of the Electronic Action Registration Form (post-registration form)**

The questions and response options registered by the healthcare professionals after the young people’s health assessment at the Youth Health Clinic.

| Electronic action registration form | |
| --- | --- |
| Choose your profession | Midwife Counsellor/psychologist  Doctor Other: |
| The visit was executed | Physically Digitally |
| The patient has an ongoing healthcare contact apart from the YHC, regarding the following health area | Physical health Mental health  Sexual health Social support (meaning emotional support and care)  No ongoing healthcare contact apart from the YHC    I do not know |
| What health areas were discussed during the present visit? | Physical health Mental health  Sexual health Social support (meaning emotional support and care) |
| Planned post-appointment | New appointment on young person’s own initiative  Planned visit to the YHC*  Young person rejects suggested re-visit at the YHC  Referral for healthcare or social care, other than the YHC |
| *Re-visit at the YNC to | Midwife Counsellor/psychologist  Doctor Other: ­­­­­­­­­­­­­­­­­­­­­­­­­­­­­­_______________________ |
