# Supplementary material for: Identification of health-related problems in youth: a mixed methods feasibility study evaluating the Youth Health Report System
Source: BMC Med Inform Decis Mak. 2024 Mar 5;24:64. doi: 10.1186/s12911-024-02465-8 (PMC10913260; doi:10.1186/s12911-024-02465-8)
Supplement: Supplementary file 3 — Supplementary Material 3 [file 12911_2024_2465_MOESM3_ESM.docx]

# **Supplementary file 3**

## **The electronic Health Report Form’s (intervention questionnaire’s) Patient-Report Outcome questionnaires and response options**

The table below describes the Patient-Report Outcome questionnaires that are included the intervention questionnaire and the response options for each Patient-Report Outcome questionnaire.

| Health area | Instrument | Questions | Response options |
| --- | --- | --- | --- |
| Mental health | General Anxiety Disorder 7-item scale (GAD-7) | The non-diagnostic instrument is aimed towards general anxiety symptoms during the past two weeks. Seven questions form a basis for conversation | The responses options are Not at all (0p) / Several days (1p) / More than half of the days (2p) / Almost every day (3).  Points are added for total score. Anxiety level threshold is recommended from 10p |
|  | Patient Health Questionnaire 9-items (PHQ-9) | The scale consists of in total nine items. Eight are used to measure symptom level of depression in the past two weeks. One item is used to measure functioning.  Can be used for depression syndrome screening, depression depth and course over time. Clinical assessment must complement use of the scale | The screening response options are Not at all (0p) / Several days (1p) / More than half of the days (2p) / Almost every day (3).  The points are added for the total score. 0-9 p = No depression, 10-14 = gray area, 15-27 = Major depression can occur.  The functioning item response options are No difficulties, Some difficulties, Great difficulties, and Extreme difficulties. |
|  | SCOFF (acronym from the questions) | The five item non-diagnostic scale maps the possibility of experiencing an eating disorder. Further clinical assessment is required. | Yes and No constitute the response options.  In case of two ”yes”-answers, further assessment and perhaps help is needed. |
| Physical health | Lifestyle habits questions from National Board of Social Affairs and health | Identifies lifestyle habits such as tobacco, exercise and eating habits by examining how often and to what extent a behavior occurs | The responses provide the basis for a conversation about lifestyle changes according to guidelines |
|  | Alcohol Use Disorder Identification Text Consumption (Audit-C) | Three items aim at detecting risky use of alcohol consumption | Scores are added. Total max score is 12. Score above 5 for men and 4 for women indicate risky use of alcohol |
|  | Health Behavior in School Children - somatic symptoms (HBSC) | Consists of six items about somatic symptoms | Response options are Rarely or never (1) / About every month (2) / About every week (3) / More than once a week (4) / About every day (5)  Scores add up  Delineation of health-related problems have been decided in collaboration with an orthopedist/psychotherapist and a neurologist with first step psychotherapy education. |
| Sexual health | Sexual health Identification Tool (SEXIT) | Assessment questionnaire followed by conversation aiming at identifying and offering advice and support for young people with risky sexual behavior, used at Youth Health Clinics | The answers generate green, yellow, and red flags, indicating risk levels, based on behavior, age and life situation. To be used in a conversation between healthcare provider and young person |
| Social support | 6-item Revised UCLA loneliness scale | Six items about the experience of loneliness and exclusion | Response options are Never (1) / Rarely (2) / Sometimes (3) / Always (4). Scores add up to a max score of 24  1-11 = No or low feelings of loneliness, 21-15 = moderate feelings of loneliness, 16-24 = high feelings of loneliness |
| Self-perceived need for behavior change to improve health | A question created by the research group to assess what is important for the young person to talk about during the conversation with a YHC healthcare provider | One item assessing what health area is prioritized by the young person to make a behavioral change | Response options are four: Physical health, Mental health, Sexual health, and Social support  The response can direct the conversation towards what meaningful behavior change for the young person |
| Behavior change | One self-efficacy question | Subjective rating of self-efficacy for self-perceived ability to perform behavior change | The response options range from 0 (not at all sure) till 100 (completely sure). Questions and response options are designed according to Bandura’s guide to constructing scales |
