# Supplementary material for: Identification of health-related problems in youth: a mixed methods feasibility study evaluating the Youth Health Report System
Source: BMC Med Inform Decis Mak. 2024 Mar 5;24:64. doi: 10.1186/s12911-024-02465-8 (PMC10913260; doi:10.1186/s12911-024-02465-8)
Supplement: Supplementary file 4 — Supplementary Material 4 [file 12911_2024_2465_MOESM4_ESM.docx]

# **Supplementary file 5**

## **Quantitative results of scientific feasibility aspect**

The data variance is portrayed in Tables S1-S8. Item responses have been summarized when possible. The total number of answers for each response option are on top of the stacks.

The primary outcome measure, the Short Warwick-Edinburgh Mental Well-Being Scale (SWEMWBS) contained in total seven questions, each with five response options, from not at all to always. When summarizing all response options, the outcome measure presented in total one missing items. Data varied over all response options (Table S1).

Table S1. Display of the Short Warwick-Edinburgh Mental Well-Being Scale data variance, all questions summarized.

The International Physical Activity Questionnaire contained three questions. In total eight missing items were reported; the first question had two missing items and the other questions reported three missing items. Data varied over the response options for all questions (Table S2).

Table S2. Display of the International Physical Activity Questionnaire data variance, all questions summarized.

The responses to the three self-efficacy questions about sexual health each reported one missing item. Most of the young people perceived their ability to protect themselves against Sexually Transmitted Disease, unwanted pregnancy and staying sober for sex, as optimal. However, data was spread over the full spectra of percent options (Table S3-S5).

Table S3. Display of data variance regarding self-efficacy for protection against Sexually Transmitted Disease.

Table S4. Display of data variance regarding self-efficacy for protection against unwanted pregnancy.

Table S5. Display of data variance regarding self-efficacy for being sober when having sex.

All seven questions in The Sense of Mastery Scale reported missing items. Questions one and two reported one missing item each, questions four, six and seven reported two missing options each and questions three and five reported three missing items each. In total, 14 out of 348 questions (4%) were missing. However, data varied over all response options (Table S6).

Table S6. Display of the Sense of Mastery Scale data variance, all questions summarized.

The eight questions in the Life and Health young questionnaire, reported no missing options. All questions reported a variety in data spread.

Table S7. Display of the Life and Health data variance, all questions summarized.

The Oslo 3-item Social Support Scale included three questions with different response options. None of the question reported missing items. Data was spread in variability over the response options (Tables S8-S10).

Table S8. Display of data variance regarding the social support item how many people are close to the respondent.

Table S9. Display of data variance regarding the social support item concerning the degree of

interest others show for the respondent.

Table S10. Display of data variance regarding the social support item how easy it is for the respondent to receive help from a neighbour.
