# Supplementary material for: Identification of health-related problems in youth: a mixed methods feasibility study evaluating the Youth Health Report System
Source: BMC Med Inform Decis Mak. 2024 Mar 5;24:64. doi: 10.1186/s12911-024-02465-8 (PMC10913260; doi:10.1186/s12911-024-02465-8)
Supplement: Supplementary file 5 — Supplementary Material 5 [file 12911_2024_2465_MOESM5_ESM.docx]

# **Supplementary file 4**

## **Qualitative results of feasibility aspects process, resources, and management**

This supplementary file includes the detailed qualitative results of the thematic analysis, regarding the process-, resources- and management feasibility aspects, with quotations for each sub-theme.

### **Feasibility of process; recruitment potential**

#### ***We knew recruitment could be hard***

The healthcare providers from the mid-size Youth Health Clinic (YHC) confirmed in the interviews, that they had previous experience of challenges to engage young people in other surveys. Therefore, the mid-size YHC healthcare professionals were concerned that the recruitment flow of young people would be slow. One problem could be that young people had the tendency to show up on the dot of the health assessment, or rather a few minutes late for their visit.

It’s a hard target group to motivate… we’ve also … developed other questionnaires and forms of surveys. To get them done is a problem. The response frequency is low – I´m afraid…

Counsellor (mid-size YHC)

The group believed that it would be easier to engage young people if they were rewarded with, for example, candy or ice cream but they also acknowledged an ethical dilemma in persuading young people to participate.

There has to be direct positive reinforcement in connection to why I should respond to anything – well over 5 minutes, I think… that they are offered something when they come to their appointment. But I don’t know, it may be a little unethical, that you should buy them to the YHC… I don’t know…

*Psychologist (mid-size YHC)*

### **Feasibility of resources; Study administration and IT-platform satisfaction**

#### ***Information and routines helped but time was an issue***

Qualitative interview data responded to the resources’ feasibility aspect, containing mid-size YHC healthcare professionals’ opinions on how they perceived the educational material, the carry through of the study and potential difficulties to carrying out a future Stepped Wedge Cluster Randomized Trial (SW-CRT) within the YHC setting. Understanding of how to use the Information Technology platform (IT-platform) was eased by the educational digital meetings, videos, and written material. The healthcare professionals found that registration of the young people in the IT-platform was a quick and effortless task to perform, once the work routines were in place.

I would have needed to get into a routine… as a reminder… like on Friday… but it’s been really quick, the actual registration.

*Psychologist (mid-size YHC)*

The healthcare professionals from the mid-size YHC pointed out challenges to extend the health assessments, to give time for young people to respond to the Electronic Health Report Form (eHRF) prototype and at the same time be able to fit their regular care into the visit. Different solutions were discussed, such as asking young people to come before their health assessment or extending the time for the health assessment, to include the eHRF prototype responding time.

… that they are booked half an hour before – that it is a booking, not just that you say: ‘you could come a little earlier’, but that the visit is from 9.30 to 11 and you take them in at 10.

*Counsellor (mid-size YHC)*

### **Feasibility of management; data accessibility, interpretation of the Electronic Case Report Form (eCRF)**

#### **The eCRF was valuable in the health assessment**

The interview data portrayed healthcare professionals’ views, from both YHCs, on the management feasibility aspect, by describing their thoughts on what to consider to further improve the eHRF prototype and eCRF, possible stressors, and what they found to be possible benefits with the eCFR.

The healthcare professionals from both YHCs were positive towards the health information in the eCRF as the basis for the health assessment and liked that the histogram colors were easily understood. They appreciated that the text responses could add details to the histograms, as an aid to understanding the result and wanted this as a default function.

I would like that – when you get the histogram – that you could see the text responses at once, to like gain depth to it…

Psychologist (mid-size YHC)

Further, they found the eHRF prototype able to reveal new information.

Well, I think this was… it gave a very clear picture of what we will talk about today..

Midwife (small-size YHC)

…then it’s good to know what issues are important to talk about and the green ones one may think that… I don’t need to talk so much about it or to take that a later time…

*Counsellor (small-size YHC)*

Also, the healthcare professionals believed that the eCRF could be used for follow-up health assessments.

Although, the healthcare professionals from the mid-size YHC were overall satisfied with how they could manage the IT-platform functions, some were not able to access the eCRF histograms, and thus used the text responses as the conversation basis.

Yes, I think I have found everything except… the eCRF.. I couldn’t… I clicked on the [icon], but I didn’t get to any histogram. That’s the only thing I didn’t manage to find my way back to. I printed the responses as text instead.

*Psychologist (mid-size YHC)*

The healthcare professionals from both YHCs, saw that using the eCRF during the appointment with young people could potentially increase their individual workload and stress during the health assessment with young people.

I do think this will lead to a whole lot of extra work, so… But it will also help us find and draw attention to the problems

*Midwife (small-size YHC)*

The psychologists and social workers from the mid-size YHC found the information from the eCRF beneficial for a general health assessment but found it more suitable for midwives than for psychologists and social workers.

We ask many of these questions at the first visit, uh, but it is helpful to see quite quickly, even before, uh, which areas you might need to prioritize if there is something that stands out and can start with that.

*Counsellor (mid-size YHC)*

I have felt that I can’t really prioritize the study over the actual conversation. And because we ask almost every question that’s [in the eHRF prototype] anyway… And I need to assess it [with the patient present].

*Psychologist (mid-size YHC)*
